# Distribution of densin in neurons

**DOI:** 10.1371/journal.pone.0205859

**Published:** 2018-10-16

**Authors:** Ayse Dosemeci, Jung-Hwa Tao-Cheng, Hannah Loo, Thomas S. Reese

**Affiliations:** 1 Laboratory of Neurobiology, National Institute of Neurological Disorders and Stroke, National Institutes of Health, Bethesda, Maryland, United States of America; 2 EM Facility, National Institute of Neurological Disorders and Stroke, National Institutes of Health, Bethesda, Maryland, United States of America; Bilkent University, TURKEY

## Abstract

Densin is a scaffold protein known to associate with key elements of neuronal signaling. The present study examines the distribution of densin at the ultrastructural level in order to reveal potential sites that can support specific interactions of densin. Immunogold electron microscopy on hippocampal cultures shows intense labeling for densin at postsynaptic densities (PSDs), but also some labeling at extrasynaptic plasma membranes of soma and dendrites and endoplasmic reticulum. At the PSD, the median distance of label from the postsynaptic membrane was ~27 nm, with the majority of label (90%) confined within 40 nm from the postsynaptic membrane, indicating predominant localization of densin at the PSD core. Depolarization (90 mM K^+^ for 2 min) promoted a slight shift of densin label within the PSD complex resulting in 77% of label remaining within 40 nm from the postsynaptic membrane. Densin molecules firmly embedded within the PSD may target a minor pool of CaMKII to substrates at the PSD core.

## Introduction

Densin, also known as densin-180, LAP1 or leucine rich repeat containing protein 7, was first identified as a 167 KDa protein enriched in the postsynaptic density (PSD) fraction [1]. Subsequent studies also characterized developmentally regulated lower molecular weight splice variants of the protein [2], [3]. Densin is abundant at the PSD, with an estimated stoichiometry of one densin to about twelve PSD-95 molecules in isolated PSD fractions [4]. Mice lacking densin show abnormal behaviors reminiscent of schizophrenia and autism type disorders [5]. A precise physiological role for densin, however, has yet to be defined.

Densin, with multiple leucine rich repeat (LRR) motifs at the N-terminal and a PDZ domain at the C-terminal, appears to be a scaffolding protein. The N-terminal LRR-containing domain targets densin to the plasma membrane [6], [3]). Binding partners of densin include CaMKII [2], [7], [8], [9], alpha-actinin [7], delta-catenin [10], Maguin-1 [11], Shanks [6] and the L-type calcium channels Cav1.2 and Cav1.3 [12], [13]. Interaction with these binding partners have suggested various functional roles for densin in neurons.

Densin has been proposed to induce dendritic branching through its interaction with delta-catenin, presumably outside the PSD. This effect of densin is antagonized by Shank, which competes with delta-catenin for binding to densin and targets densin to the PSD [6]. Densin at the PSD, in turn, has been proposed to anchor and/or position CaMKII [2], [7], [5]. Recent studies revealed interaction of densin with L-type calcium channels. Densin has been reported to simultaneously bind Cav1.3 and CaMKII thereby promoting facilitation of the channel [12]. Densin has also been shown to bind to Cav1.2 type channels resulting in an increase in Cav1.2 surface expression and calcium currents [13]. Although these studies show that densin can interact with, and regulate calcium channels, it is not clear whether the native proteins actually co-localize, and if they do so, where.

In the present study we examine the distribution of endogenous densin within neurons at the ultrastructural level by immuno-electron microscopy. Concentration of densin at the core of the PSD implies a role in postsynaptic organization and function. The presence of densin on somal-dendritic plasma membranes outside the PSD supports additional extrasynaptic roles.

## Materials and methods

The animal protocol was approved by the National Institute of Neurological Disorders and Stroke/ National Institute of Deafness and Communication Disorders/National Center for Complementary and Integrative Health Animal Use and Care Committee and conforms to NIH guidelines. Approval #Asp 1159–15.

### Antibodies

Two antibodies were used for detection of densin. Antibody 1 was a mouse monoclonal antibody (Clone G-1) raised against a peptide corresponding to AAs 596–895 from Santa Cruz Biotechnology (1:100 for Western, 1:50 for immunoEM). Antibody 2 was a rabbit polyclonal antibody raised against a peptide corresponding to AAs 432–562 of rat densin from Novus Biologicals. (1:100 for Western, 50–100 for immunoEM).

### Preparation of subcellular fractions from rat brain and western immunoblotting

Brains from adult rats (7–12 weeks of mixed gender) were supplied by Rockland Immunochemicals Inc. (Gilbertsville, PA). Animals were anesthetized with isoflurane, brains were collected within two minutes after cervical dislocation and were immediately frozen in liquid nitrogen. Brains were rapidly thawed in isotonic sucrose solution and dissected to remove white matter. Cerebral cortices were homogenized in isotonic sucrose. Subcellular fractionation was essentially as described previously [14]. Homogenates were centrifuged at 900 g for 10 min and pellets (P1) containing nuclei were discarded. The supernatants (S1) were centrifuged at 10,500 g for 12 min to obtain P2 and S2 fractions. S2 fraction was further centrifuged 82,700 g for 2 h to obtain P3 (light membranes) and S3 (cytosolic) fractions. P2 was fractionated on a sucrose gradient to isolate synaptosomes. Synaptosomes were treated with 0.5% TritonX-100 and the detergent insoluble pellet was fractionated on a sucrose gradient. Crude PSD fraction from the 1.5/2.1M sucrose interface was extracted with 0.5% TritonX-100, 75 mM KCl and collected on a sucrose cushion. Proteins from subcellular fractions were resolved by SDS-PAGE using 4–15% Mini-PROTEAN TGX Precast polyacrylamide gels (BioRad). Gels were transferred to PVDF membranes using the Trans-Blot Turbo Transfer System (BioRad), blocked, incubated with primary and secondary antibodies, and visualized via chemiluminescence.

### Preparation and treatment of hippocampal cultures

All animals were housed in an NIH intramural research program vivarium as described previously [15]. Nine Sprague Dawley timed pregnant rats from Taconic Farms (Germantown, MD, USA) and Charles River (Raleigh, NC, USA) were used. Pregnant dams were killed by CO_2_ inhalation. Embryos were then collected by caesarian section and decapitated with sharp scissors (Animal protocol Number:ASP1159).

Cell cultures were prepared as previously described [15]. Briefly, hippocampi from embryonic 20 days-old rat fetuses were dissociated by papain, and then plated onto rat glial feeder cultures. Cultures were maintained in MEM Eagle Salts and kept in 10% CO_2_ incubator at 35°C, and experiments were carried out with three weeks-old cultures.

Culture dishes were placed on a floating platform in a water bath maintained at 37°C. Control incubation medium was: 124 mM NaCl, 2 mM KCl, 1.24 mM KH_2_PO_4_, 1.3 mM MgCl_2_, 2.5 mM CaCl_2_, 30 mM glucose in 25 mM HEPES at pH 7.4. High K^+^ medium was at 90 mM KCl, with osmolarity compensated by reducing the concentration of NaCl. For high K^+^ experiments, cell cultures were washed with control medium and treated for 2 min with either control or high K^+^ media, and then fixed immediately. This condition (2 min treatment with 90 mM K+) was previously shown to elicit significant morphological changes and molecular re-organization at the PSD (review: [16]).

### Pre-embedding immunogold labelling and electron microscopy

All steps were carried out at room temperature unless otherwise indicated. Cell cultures were fixed with paraformaldehyde (EMS, Fort Washington, PA) in PBS, washed and stored in PBS at 4°C for immunolabeling. Optimal fixation conditions using antibody 1 and antibody 2 were: 2% paraformaldehyde for 15 min and 4% paraformaldehyde for 35 min respectively. Fixed samples were permeablized and blocked with 0.1% saponin and 5% normal goat serum in PBS for 30 min, or permeablized with 50% ethanol for 10 min and then blocked with 5% normal goat serum in PBS for 20 min. Samples were then incubated with primary antibodies for 1 hr, washed and incubated with secondary antibodies (Nanogold from Nanoprobes, Yaphand, NY) for 1 hr, washed and fixed with 2% glutaraldehyde in PBS, stored in fixative at 4°C until ready for silver enhancement. Samples were washed in deionized water, then silver enhanced (HQ kit from Nanoprobes), treated with 0.2% osmium tetroxide in 0.1 M phosphate buffer at pH 7.4 on ice for 30 min, followed by treatment with 0.25% uranyl acetate in 0.1 N acetate buffer at pH 5.0 at 4°C for 1 hour, then dehydrated through a graded series of ethanol and finally embedded in epoxy resins. Controls for antibody specificity included omitting primary antibodies and using different primary antibodies as controls for each other (S1 and S2 Figs).

Thin sections of 70–90 nm were cut *en face* and picked up on 400 mesh grids, and counter-stained with uranyl acetate and lead citrate. Images were taken on a JEOL 1200 EX electron microscope with a digital CCD camera (AMT XR-100, Danvers, MA, USA) at 10, 000 times magnification for plasma membranes and at 40, 000 times magnification for PSDs.

### Morphometry

At least 4–5 grid openings were randomly chosen from each thin-sectioned sample and every cross-sectioned asymmetric synapse encountered was photographed for morphometry. The PSD region was marked as the area extending 120 nm beneath the postsynaptic membrane [17]. The distance of each gold particle from the postsynaptic membrane within the PSD was measured. Segments of clearly cross-sectioned postsynaptic membranes were marked and the distance of each gold particle within the area underneath the segment was measured from the center of the gold particle to the outside edge of the postsynaptic membrane. All distance measurements from a sample were plotted in a histogram to examine the laminar distribution of label from the postsynaptic plasma membrane. Statistical significances of the differences in median distance of label from the postsynaptic membrane was assessed by the Wilcoxon test. Labeling intensity at the PSD is defined as number of gold particles within the 120 nm depth of the PSD, divided by the length of the PSD (per μm). Statistical significance of the differences in labeling intensity between groups was assessed by Student’s t test.

## Results

**Fig 1:**
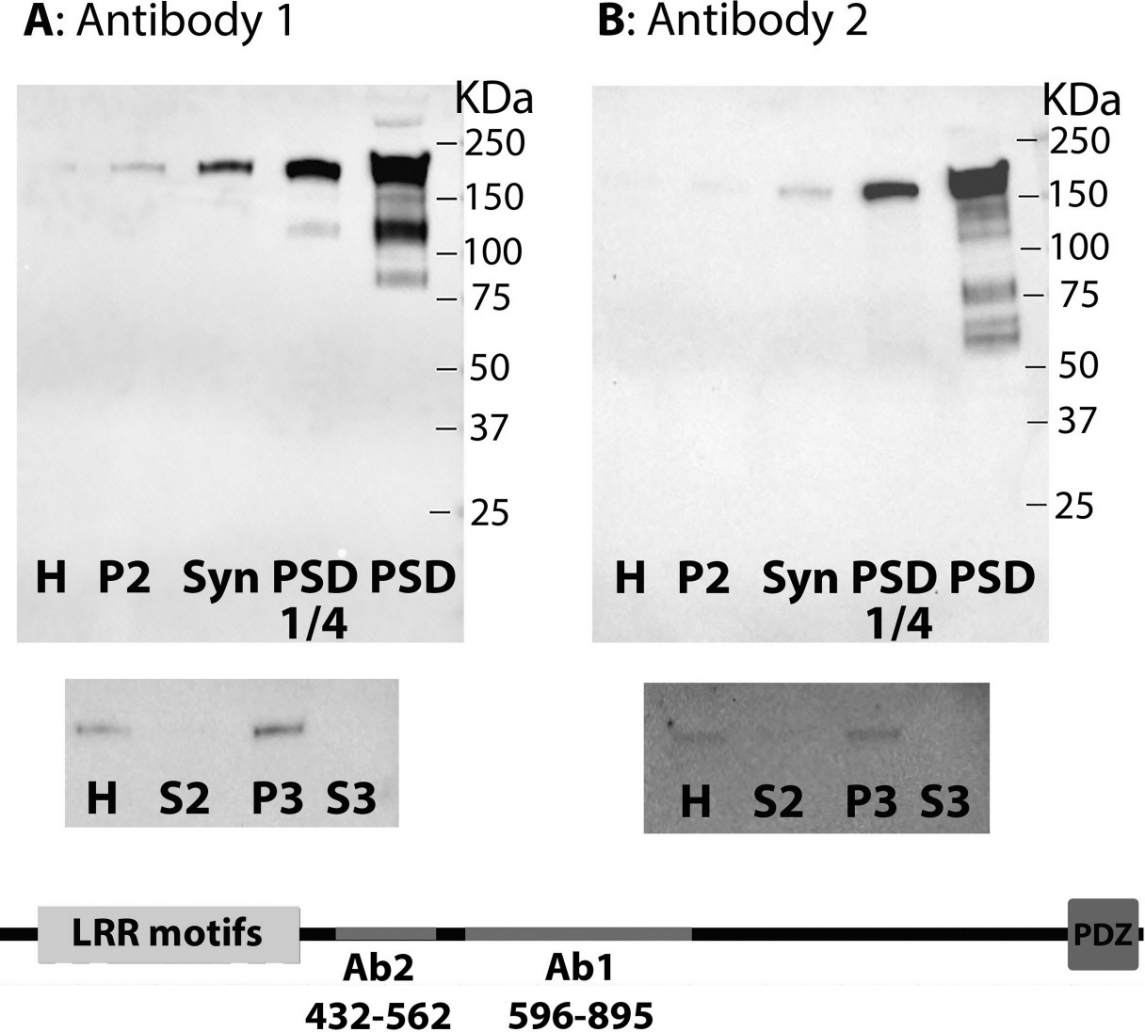
Distribution of densin in subcellular fractions from rat cerebral cortex. Western immunoblots using antibody 1 (**A**) or antibody 2 (**B**) show enrichment of densin in the PSD fraction. All lanes contain 20μg of protein except ‘PSD ¼’ which contains 5 μg. Last lanes on the right: signal at the major ~180 KDa band is over-saturated allowing visualization of the lower molecular weight bands. H (homogenate), P2 (pellet after 10,500gX12min centrifugation), Syn (synaptosome), PSD (postsynaptic density). Middle panels: A slight enrichment in the light membrane fraction (P3) as compared to homogenate is observed, while the cytosol fraction (S3) does not show detectable signal. Bottom: Graphical depiction of densin molecule with LRR motifs at the N-terminal and a PDZ domain at the C-terminal. The peptide sequences used to generate antibody 1 (Ab1) and antibody 2 (Ab2) are located in the middle region between these N-and C-terminal domains.

Levels of densin in subcellular fractions from adult rat cerebral cortex were compared by Western immunoblotting (Fig 1). Two antibodies directed to non- overlapping sequences in the middle region of the densin molecule were used (Fig 1 bottom). Both antibodies recognized a major band between 150 KDa and 250 KDa (Fig 1 top) that seems to correspond to the ~180 KDa adult form of densin [1], [3]. Sets of minor lower molecular weight bands that become visible only after the signal from the main band is over-saturated (PSD lanes on the right) appear to be distinct for each antibody and may correspond to different splice variants described previously [3]. Alternatively, these lower molecular weight bands may correspond to proteins other than densin. Antibody 1 and antibody 2 revealed a similar distribution of the ~180 KDa main band in subcellular fractions, with a drastic enrichment in the PSD fraction. A slight enrichment of the ~180 KDa band in the P3 fraction (light membranes devoid of PSD) compared to homogenate (Fig 1 middle) suggests that densin is also present on membranes outside the PSD.

**Fig 2:**
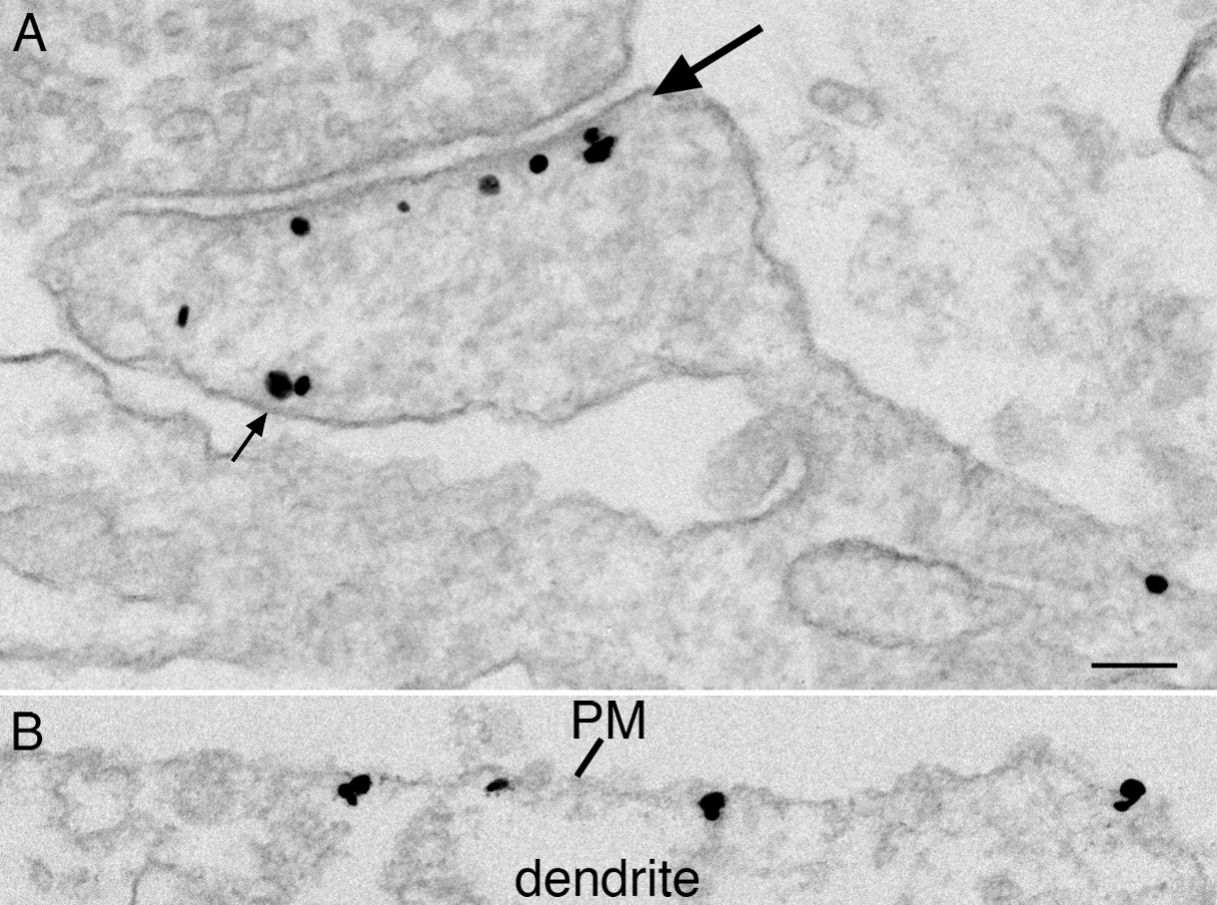
Densin immunolabel is concentrated at the PSD. Electron micrographs showing immunogold labeling of cultured hippocampal neurons using densin antibody 1. Silver-enhanced gold particles are seen as dark grains of heterogeneous size **A**: excitatory synapse showing intense labeling at the PSD (large arrow) with some labeling on the plasma membranes near the synapse (small arrow). **B**: dendritic region showing scattered labelling on the plasma membrane. Scale bar = 0.1 μm.

The two densin antibodies tested by Western immunoblotting were subsequently used for immunogold labeling of hippocampal neuronal cultures. Antibody 1 promoted selective, intense labeling of PSDs (Figs 2A and S3) with more than 90% PSDs labeled. Also, less intense, scattered labeling was observed on extrasynaptic plasma membranes throughout the soma and dendrites (Figs 2B and S3) and sometimes near synapses (Fig 2A small arrow).

**Fig 3:**
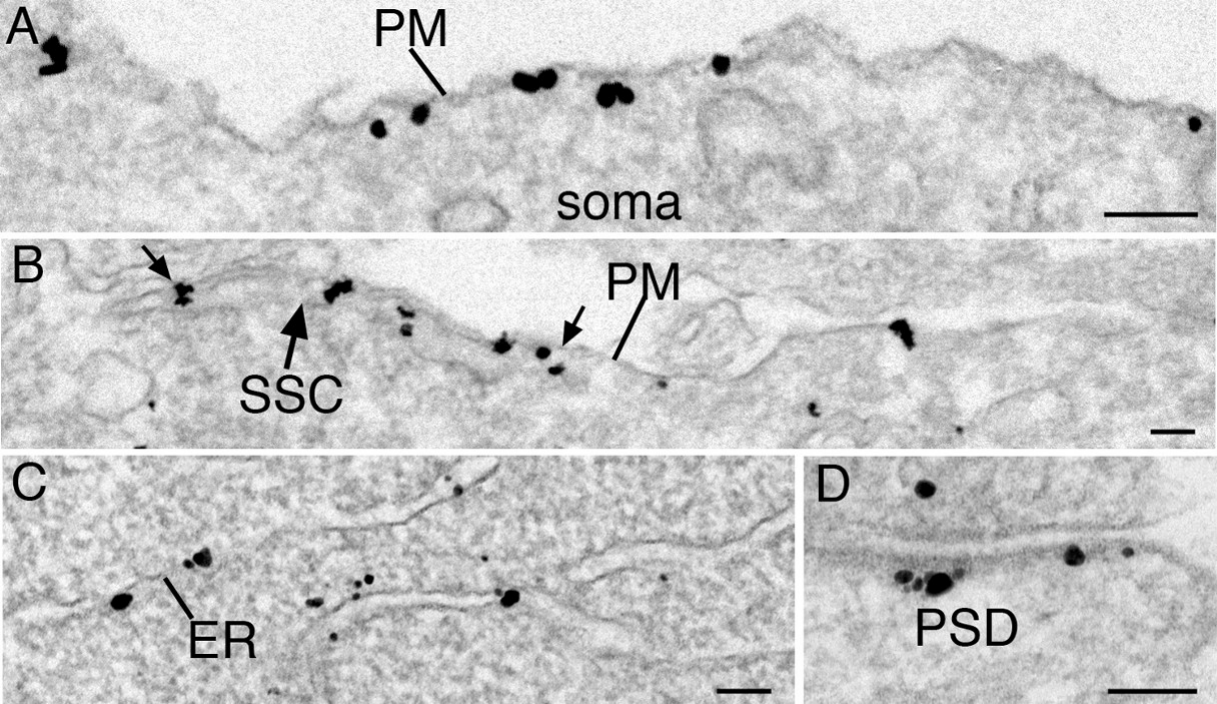
Densin immunolabel on extrasynaptic membranes. Electron micrographs showing immunogold labeling of cultured hippocampal neurons using densin antibody 2. Labeling on plasma membranes (PM, A, B) and ER (C) as well as on PSDs (D) is detected. Subsurface cisterns (SSC) were occasionally observed under patches of plasma membrane with a cluster of densin label (area bordered by the two small arrows). Scale bar = 0.1 μm.

In general, antibody 2 produced a similar pattern of selective labeling at the PSD (Figs 3D and S4), but at a lower labeling efficiency compared to antibody 1, with only about half of PSDs labeling. On the other hand, antibody 2 appeared to be more effective in labeling extrasynaptic membranes, including somatodendritic plasma membranes (Figs 3A, 3B and S4) and ER (Figs 3C and S4). Interestingly, the plasma membrane atop subsurface cisterns (SSC, specialized compartments of ER in close apposition with the plasma membrane [18]) was occasionally observed to contain a cluster of concentrated label (Fig 3B, area between two small arrows).

**Fig 4:**
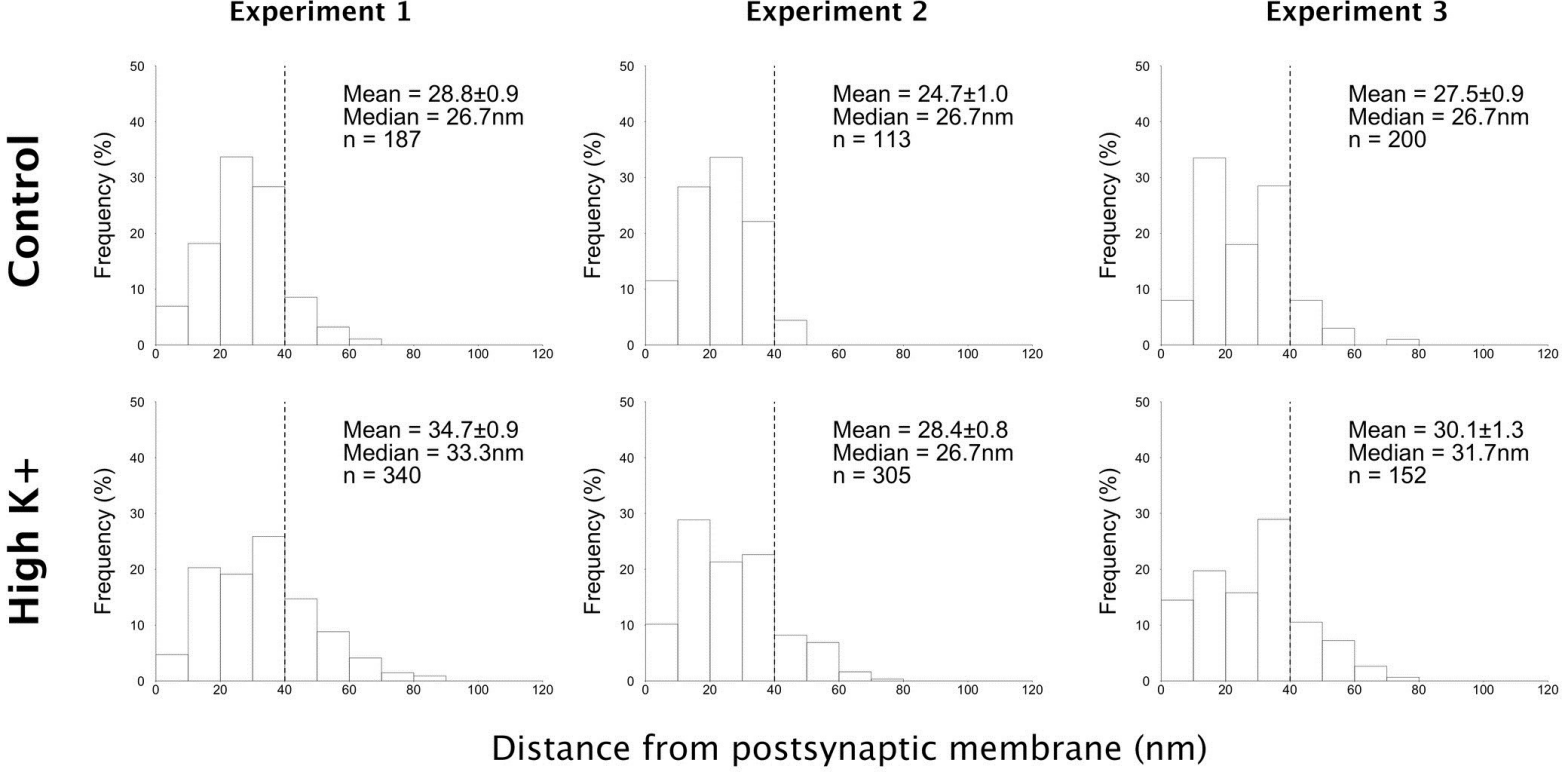
Distribution of Densin label within the PSD. Distances of gold particles from the postsynaptic membrane were measured in three experiments. Histograms show the percentage of label in consecutive laminar layers (10 nm bins). Under basal (control) conditions 90% of densin label is confined within the PSD core (40 nm from the postsynaptic membrane, marked by a dashed line on histograms). Following depolarization with high K^+^ (90 mM for 2 min) a slight shift of label towards the PSD pallium (40–120 nm from the postsynaptic membrane) was observed, with 77% of label remaining within the PSD core.

Of the two antibodies tested, antibody 1 promoted a higher labeling efficiency of PSDs and thus was used in further experiments to document the localization of densin within the PSD by electron microscopy. The PSD is made up of two layers: an electron dense ‘PSD core’ with a prominent PSD-95 scaffold; and a deeper ‘PSD pallium’, with a scaffold comprised of Shanks and Homer [16]. For quantification purposes, the boundary between the two layers was set at 40 nm from the postsynaptic membrane [19]. The distances of gold particles from the postsynaptic membrane were measured in three experiments to accurately characterize the position of densin molecules within the PSD complex, (Fig 4). Under basal (control) conditions, the median distance of densin label from the postsynaptic membrane was ~27 nm, with 90%, of gold particles confined within the PSD core, the area extending 40 nm deep from the postsynaptic membrane (Fig 4, upper panels).

Following depolarization with high K^+^ for 2 min, the median distance of densin label from the postsynaptic membrane recorded a small shift from 27 nm to 31 nm (the differences did not reach statistical significance in individual experiments). Percent label within 40 nm of the postsynaptic membrane changed from 90 ± 2.6% in control to 77 ± 4.0% following high K^+^ (P<0.05 paired t-test, Fig 4, lower panel). The distance analysis of densin label indicates that the protein is mostly embedded in the PSD core (Fig 4) with only a slight outward shift of densin label away from the postsynaptic membrane upon depolarization.

When the overall labeling density for densin at the PSD (PSD core and PSD pallium) was compared under control and depolarization conditions, no consistent trend was detected (Table 1). A statistically significant increase was found in one experiment but the other two experiments showed insignificant decrease and increase, respectively. This inconsistency could be contributed by differences between individual batches of hippocampal cultures, or immunolabeling technical issues. Notably, the mild fixation conditions tolerated by this antibody can easily result in variable detection issues from sample to sample. In sum there was no significant decrease in the concentration of densin at the PSD upon depolarization.

**Table 1:** Density of label for densin at the PSD (number of gold particles per μm PSD).

		Control	High K^+^	% K^+^/cont	Student’s t-test	Wilcoxon test
Exp 1	mean±SEM (n)	16.2 ± 1.2 (50)	14.2 ± 0.8 (80)	88%	N. S.	
	median	14.4	12.8			N. S.
	range	5.0–38.7	3.8–33.9			
Exp 2	mean±SEM	17.6 ± 1.6 (32)	26.6 ± 1.4 (57)	150%	P<0.0001	
	median	16.1	28.0			P<0.0001
	range	3.6–44.7	4.3–49.5			
Exp 3	mean±SEM	21.4 ± 1.6 (36)	24.5 ± 1.6 (34)	114%	N. S.	
	median	23.5	22.9			N. S.
	range	5.0–36	8.4–43.6			

(n) = number of synapses measured; N. S. = non-significant.

Differences of means between control and high K^+^ from 3 experiments are non-significant by t-test.

## Discussion

Biochemical and ultrastructural approaches both indicate that densin is most concentrated at the PSD. Immuno-electron microscopy provides more detailed information on the position of densin *within* the PSD. Label for densin using antibody 1 lies at the same average distance from the postsynaptic membrane as that for PSD-95 [19] (antibody against a sequence between PDZ2 and PDZ3 domains), the main scaffold protein at the PSD core. These data, combined with previously reported information on the association partners at the N- and C-termini of densin, allows assessment of the orientation of the molecule within the PSD. Similar to other LAP (LRR and PDZ domain) proteins, the N-terminal LRR-containing section of densin is likely to be interacting with the membrane [6]. The present data implies that the middle portion of densin, recognized by the antibody used, is also located at the PSD core, at an average distance of 27 nm from the postsynaptic membrane. Since the C-terminus of densin is known to interact with Shanks [6], this part of the protein must extend to the deeper region, called the PSD pallium, where the Shank/homer scaffold is located [16] [20]. Thus, combined data from several studies suggest that densin, like PSD-95 [21], must be spanning the whole thickness of the PSD core, from the postsynaptic membrane to the PSD pallium.

The densin pool at the PSD seems to be stable following 2 min depolarization in high K^+^, a condition known to induce substantial changes in distributions of other PSD proteins [16]. No statistically significant decrease in the label density of densin was detected in any of the three experiments following depolarization. While depolarization was observed to induce a small shift in the laminar position of densin label, we think such a small shift is more likely due to a change in molecular conformation of densin rather than a movement of the whole protein.

The peptide sequence (aa 596–895) used to produce antibody 1 encompasses the binding site of densin for activated CaMKII, the so-called densin-IN domain (aa 793–824, [9]). Thus, the observed distribution of the label with antibody 1 implies that CaMKII associates with the central domain of densin within the PSD core. While the majority of CaMKII molecules at the PSD appear to be located at a deeper region [22] designated as the PSD pallium [16]), immuno-electron microscopy studies on perfusion-fixed brain tissue [22], as well as tomographic analysis of isolated PSDs [23], reveal a minor pool of CaMKII closer to the cleft side. It is possible that this minor pool of CaMKII at the PSD core is associated with densin.

The PSD is a tightly organized complex where protein-protein associations severely restrict free diffusion of proteins. Thus, phosphorylation of a CaMKII substrate at the PSD would probably require a dedicated CaMKII molecule strategically targeted towards the phosphorylation site. We speculate that binding of activated CaMKII to the central portion of densin could position the kinase to promote phosphorylation of specific substrates located at the PSD core, including, SynGAP and AIDA-1. Indeed, both of these molecules are located at the PSD core under resting conditions, but move out under excitatory conditions, contingent on the activation of CaMKII [24], [25].

Recent studies revealed a role for densin in the regulation of L-type calcium channels, Cav1.3 and Cav1.2 [12], [13]. Densin binds both Cav1.3 and CaMKII to form a complex that results in facilitation of the channel [12]. Densin binding to Cav1.2 increases Cav1.2 surface expression [13]. However, although L-type calcium channels can be present at dendritic spines, unlike densin, they appear to be excluded from PSDs. Indeed, immunofluorescence microscopy shows that PSD-95 and CaV1.2 signals don’t overlap within spines [26] and immuno-electron microscopy studies show a lack of labelling of PSDs for CaV1.2 as well as CaV1.3 [27], [28]. These observations suggest that the pool of densin firmly embedded within the PSD core is unlikely to associate with L-type calcium channels. The present study by immuno-electron microscopy indicates an additional presence of densin at plasma membranes, including those near synapses. Thus, it is likely that densin interacts with L-type calcium channels on plasma membranes outside the synapse. This proposition is in agreement with the finding that Cav1.3-Densin- CaMKII complexes are immunoprecipitated from TX-100 soluble fractions [12] that should not contain detergent-insoluble PSDs.

In conclusion, the present immunoEM study shows a concentration of densin label at the PSD core and some additional labeling on extrasynaptic plasma membranes. We speculate that densin at the PSD can target active CaMKII to substrates such as SynGAP and AIDA-1 located at the PSD core. Extrasynaptic densin may simply be a reservoir for the PSD pool or may have distinct functions such as anchoring and regulating extrasynaptic L-type Ca2+ channels.

## Supporting information

S1 Fig Specific labeling of the PSD with densin ab1. Images are from zonula radiatum of the CA1 region of hippocampus of perfusion-fixed mouse brain. The postsynaptic density (PSD) is preferentially labeled using ab1 (arrows in A, B), whereas no label on PSDs is observed when the primary antibody is omitted (C, D). Scale bar = 0.1 μm.
(PDF)

S2 Fig Specific labeling of the PSD with densin ab2. Images are from sister cultures of dissociated hippocampal neurons processed in parallel. PSDs are labeled with densin ab2 (arrows in A, B), but not when using another primary antibody (polyclonal rabbit against a synthetic peptide DIEVLQEQIRC) (arrows in C, D). Scale bar = 0.1 μm.
(PDF)

S3 Fig Low magnification images show labeling at the PSD and dendritic plasma membranes using densin ab1. Label for densin ab1 is specifically concentrated at the PSD (large arrows in A, B, C) and scattered on dendritic plasma membranes (small arrows in A-D). Plasma membrane of presynaptic terminals (T in A-C) are not labeled. Scale bar = 0.1 μm.
(PDF)

S4 Fig Low magnification images show labeling at the PSD, plasma membrane of neuronal soma, and the endoplasmic reticulum using densin ab2. Label for densin ab2 is specifically concentrated at the PSD (large arrows in A, C). Plasma membrane of neuronal somas (PM in B, D) and the endoplasmic reticulum (ER in B, D) are also labeled. Scale bar = 0.1 μm.
(PDF)

S5 Fig Histograms of labeling density for densin at the PSD. Measurements of density of label for densin at the PSD under control (top row) and depolarization (lower row) conditions. Mean and median values and statistical analysis are summarized in Table 1.
(PDF)
