# Supplementary material for: Distribution of densin in neurons
Source: PLoS One. 2018 Oct 16;13(10):e0205859. doi: 10.1371/journal.pone.0205859 (PMC6191147; doi:10.1371/journal.pone.0205859)
Supplement: S1 Fig — Images are from zonula radiatum of the CA1 region of hippocampus of perfusion-fixed mouse brain. The postsynaptic density (PSD) is preferentially labeled using ab1 (arrows in A, B), whereas no label on PSDs is observed when the primary antibody is omitted (C, D). Scale bar = 0.1 μm. (PDF) [file pone.0205859.s001.pdf]

S1 Fig. Specific labeling of the PSD with densin ab1.

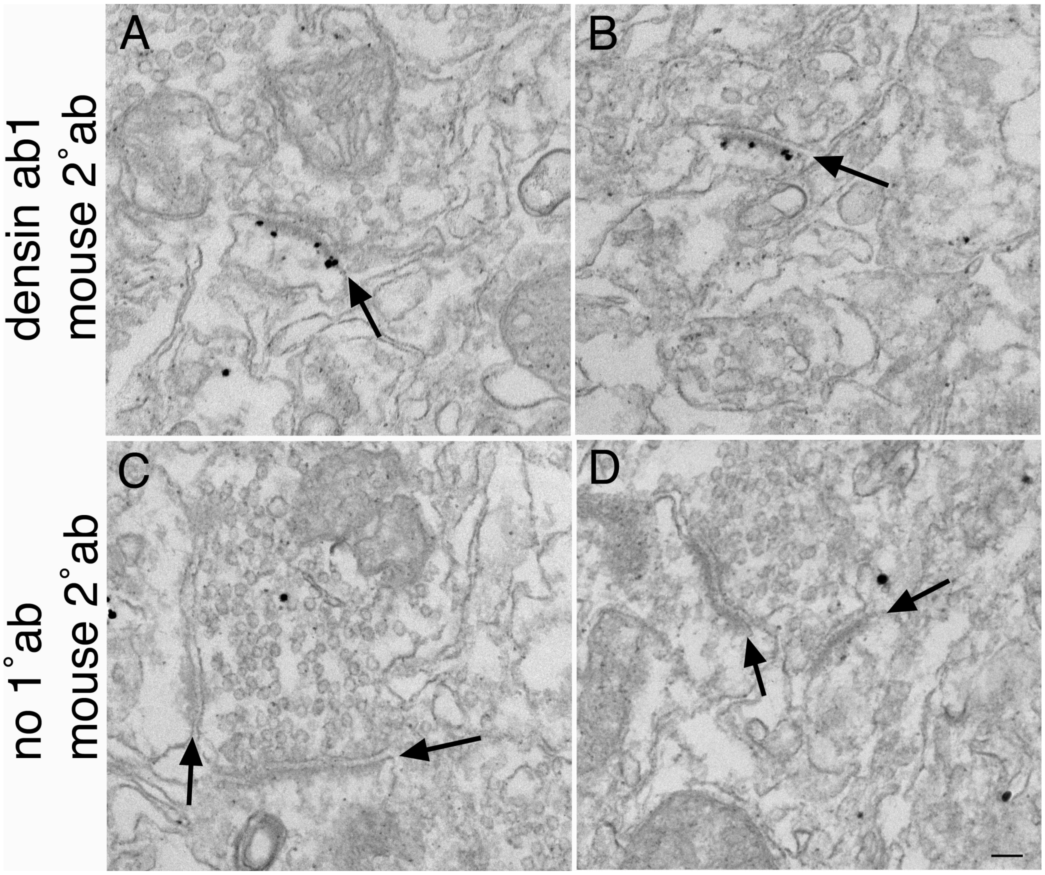

Images are from zonula radiatum of the CA1 region of hippocampus of perfusion-fixed mouse brain. The postsynaptic density (PSD) is preferentially labeled using ab1 (arrows in A, B), whereas no label on PSDs is observed when the primary antibody is omitted (C, D). Scale bar = 0.1 µm.
