# Supplementary material for: Distribution of densin in neurons
Source: PLoS One. 2018 Oct 16;13(10):e0205859. doi: 10.1371/journal.pone.0205859 (PMC6191147; doi:10.1371/journal.pone.0205859)
Supplement: S2 Fig — Images are from sister cultures of dissociated hippocampal neurons processed in parallel. PSDs are labeled with densin ab2 (arrows in A, B), but not when using another primary antibody (polyclonal rabbit against a synthetic peptide DIEVLQEQIRC) (arrows in C, D). Scale bar = 0.1 μm. (PDF) [file pone.0205859.s002.pdf]

S2 Fig. Specific labeling of the PSD with densin ab2.

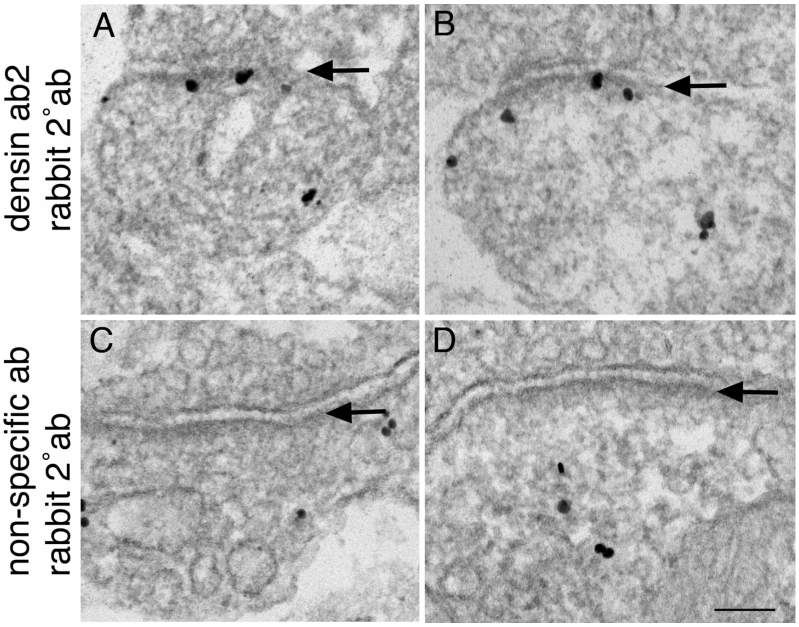

Images are from sister cultures of dissociated hippocampal neurons processed in parallel. PSDs are labeled with densin ab2 (arrows in A, B), but not when using another primary antibody (polyclonal rabbit against a synthetic peptide DIEVLQEQIRC) (arrows in C, D). Scale bar = 0.1 μm.
