# Supplementary material for: Distribution of densin in neurons
Source: PLoS One. 2018 Oct 16;13(10):e0205859. doi: 10.1371/journal.pone.0205859 (PMC6191147; doi:10.1371/journal.pone.0205859)
Supplement: S3 Fig — Label for densin ab1 is specifically concentrated at the PSD (large arrows in A, B, C) and scattered on dendritic plasma membranes (small arrows in A-D). Plasma membrane of presynaptic terminals (T in A-C) are not labeled. Scale bar = 0.1 μm. (PDF) [file pone.0205859.s003.pdf]

S3 Fig. Low magnification images show labeling at the PSD and dendritic plasma membranes using densin ab1.

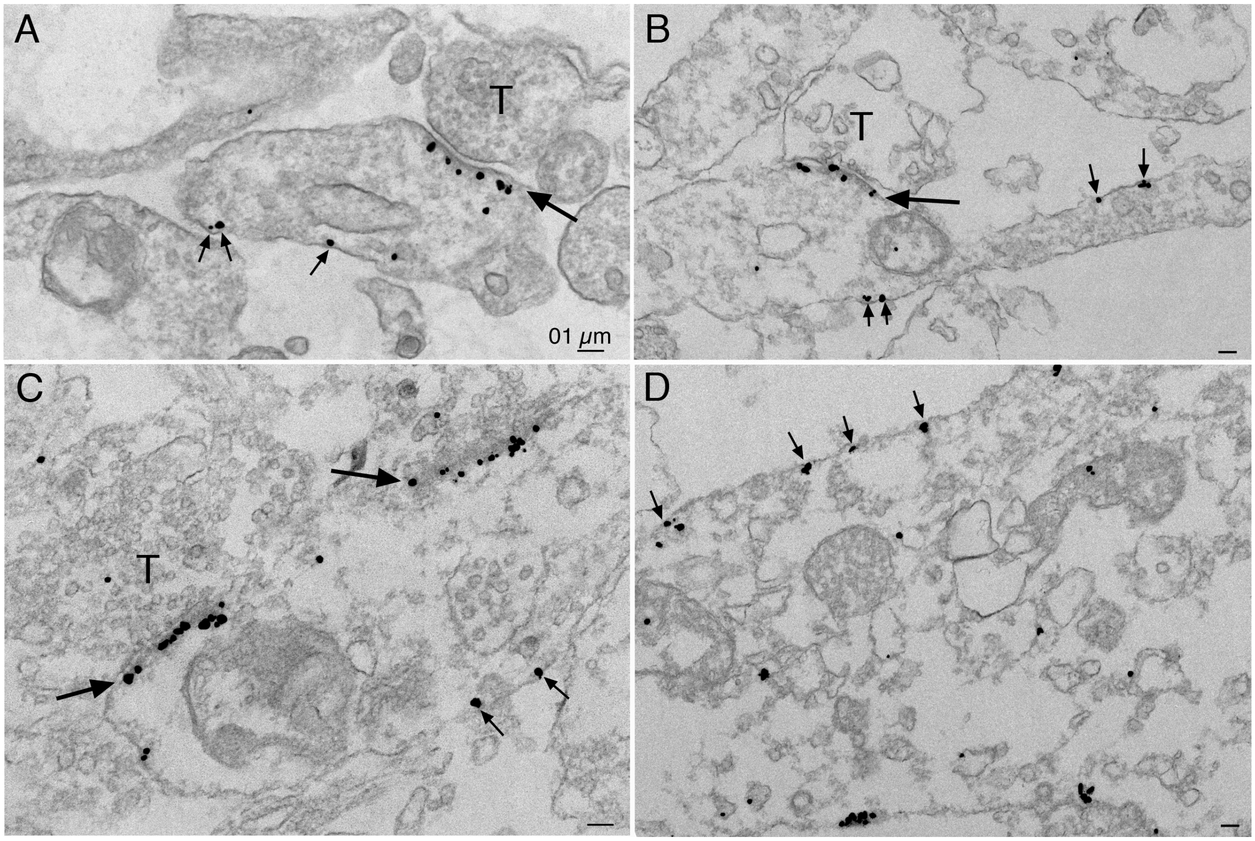

Label for densin ab1 is specifically concentrated at the PSD (large arrows in A, B, C) and scattered on dendritic plasma membranes (small arrows in A-D). Plasma membrane of presynaptic terminals (T in A-C) are not labeled. Scale bar = 0.1 μm.
