# Supplementary material for: Distribution of densin in neurons
Source: PLoS One. 2018 Oct 16;13(10):e0205859. doi: 10.1371/journal.pone.0205859 (PMC6191147; doi:10.1371/journal.pone.0205859)
Supplement: S4 Fig — Label for densin ab2 is specifically concentrated at the PSD (large arrows in A, C). Plasma membrane of neuronal somas (PM in B, D) and the endoplasmic reticulum (ER in B, D) are also labeled. Scale bar = 0.1 μm. (PDF) [file pone.0205859.s004.pdf]

S4 Fig. Low magnification images show labeling at the PSD, plasma membrane of neuronal soma, and the endoplasmic reticulum using densin ab2.

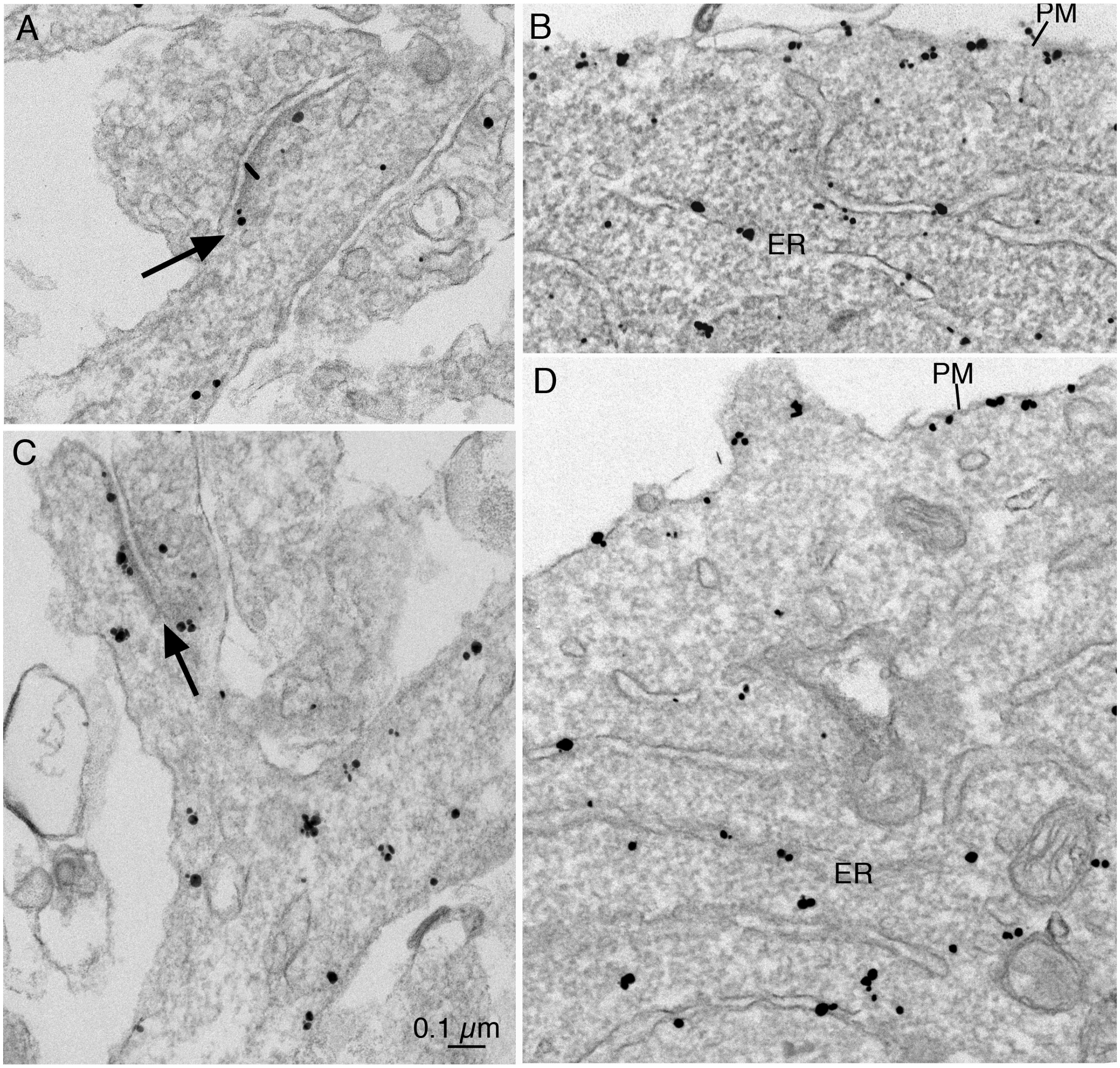

Label for densin ab2 is specifically concentrated at the PSD (large arrows in A, C). Plasma membrane of neuronal somas (PM in B, D) and the endoplasmic reticulum (ER in B, D) are also labeled. Scale bar = 0.1 µm.
