# Supplementary material for: Distribution of densin in neurons
Source: PLoS One. 2018 Oct 16;13(10):e0205859. doi: 10.1371/journal.pone.0205859 (PMC6191147; doi:10.1371/journal.pone.0205859)
Supplement: S5 Fig — Measurements of density of label for densin at the PSD under control (top row) and depolarization (lower row) conditions. Mean and median values and statistical analysis are summarized in Table 1. (PDF) [file pone.0205859.s005.pdf]

S5 Fig. Histograms of labeling density for densin at the PSD.

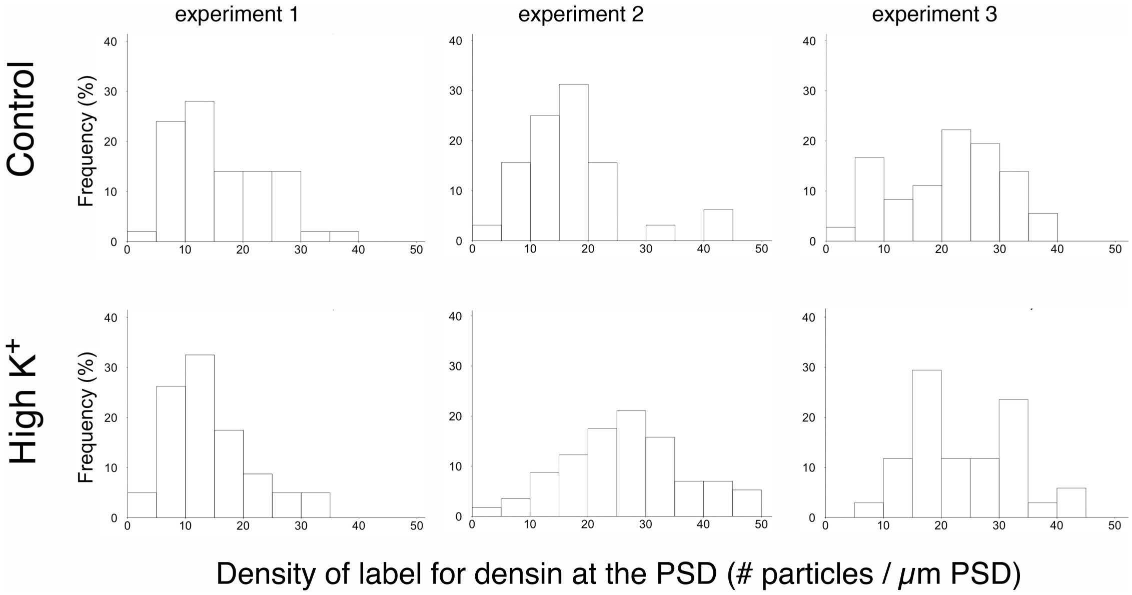

Measurements of density of label for densin at the PSD under control (top row) and depolarization (lower row) conditions. Mean and median values and statistical analysis are summarized in Table 1.
